# The digital competence of academics in higher education: is the glass half empty or half full?

**DOI:** 10.1186/s41239-022-00376-0

**Published:** 2023-02-03

**Authors:** Andreia Inamorato dos Santos, Ernesto Chinkes, Marco A. G. Carvalho, Claudia M. V. Solórzano, Lilian S. Marroni

**Affiliations:** 1 Joint Research Centre, European Commission, Seville, Spain; 2 Universidad de Buenos Aires, Buenos Aires, Argentina; 3 University of Campinas, Limeira, Brazil; 4 Instituto Politécnico Nacional, Mexico City, Mexico; 5 Federal Institute of Education, Science and Technology of São Paulo, Piracicaba, Brazil

**Keywords:** Lifelong learning, Digital literacy, DigCompEdu, Professional development, ICT

## Abstract

This paper aims to evaluate and discuss the digital competence of academics at universities, to identify challenges and define recommendations for policy. This study was conducted through collaboration between the Joint Research Centre (JRC) of the European Commission and Metared of the Universia Foundation, surveying 30,407 participants who present the perceptions of their own digital competence levels. These self-reflections took place in universities in seven countries, namely Argentina, Brazil, Colombia, Chile, Peru, Mexico and Portugal, and used the Check-In tool, which consists of 22 questions based on the European Framework for the Digital Competence of Educators—or ‘DigCompEdu’ framework. A descriptive statistical analysis was performed, followed by a qualitative evaluation. Almost 70% of the academics have an average intermediate level of competence when the data is aggregated, with results varying in each DigCompEdu area according to the specific question asked. There is no significant difference between young and senior academics, as well as between men and women. The results present a discussion of whether the age and gender of educators and their work environment have an impact on their digital competence level, and at the same time highlights the areas in which educators perceive themselves to be most and least competent. It shows how the amount of institutional support that is offered affects the academics’ perceptions of their level of digital competence. On the basis of the results, recommendations are presented for higher-education institutions, with the aim of supporting the professional development of their academics.

## Introduction

The rapid advances in information and communication technology (ICT) have created new conditions and challenges for the information society. In higher education, these challenges are related to the use of such technologies in preparing students for life and for the changing realities of the labor market.

In this sense, ICT is crucial for the development of generic competences within academic curricula, as well as for supporting cultural interpretation and integration (Guillén-Gámez & Mayorga-Fernández, 2020). Digital technologies allow us to expand the possibilities for generating knowledge, sharing it and disseminating it in spaces that promote digital empowerment.

The use of technological advancements in teaching has transformed the practices of literacy (Cabero-Almenara et al., 2020) and traditional methodologies. It has become essential for teachers when designing innovative solutions to real-world problems (Guillén-Gámez & Mayorga-Fernández, 2020), and has consequently increased student motivation (Laskaris et al., 2017).

New educational policies and documents introduced in various countries demonstrate the importance given to technology and digital-related capabilities (Spante et al., 2018). For example, one of the strategic objectives of the European Commission in the field of education and training is to encourage innovation and creativity, promoting the acquisition of transversal competences, including digital competence, by all citizens (García et al., 2020). According to the Council of the European Union (European Union, 2018), digital competence is one of eight essential competences for lifelong learning (the other seven are literacy; multilingual competence; STEM; citizenship; entrepreneurship; social competence and learning to learn; and cultural awareness and expression).

Digital competence is a concept largely introduced by European policy documents, having then emerged as a research topic of the moment (Zhao et al., 2021). It was introduced by Ferrari (2013) with the publication of the DigComp framework, which served as basis for several other digital competence frameworks developed by the European Commission, such as DigComp for Consumers (2016), DigCompOrg (2017), DigCompEdu (2017), and the newest version DigComp 2.2 (2022), to cite a few.

Digital competence is thus considered an essential skill for academics, supporting them in the management of several technological, curricular and pedagogical aspects (Punya Mishra & Koehler, 2008). Academics’ digital competence relates to digital skills, pedagogic-didactic awareness and the understanding of the impact of learning strategies on students’ learning (Krumsvik, 2009). As a facilitator of the learning process, the academic also supports the development of digital competences by their students and peers.

In the context of teaching and learning in higher education, digital teaching competences go beyond the critical and responsible use of ICT resources, because other relevant skills concerning evaluation, cooperation and feedback to students are also necessary as part of academics’ habitual educational praxis in the digital world (Cabero-Almenara et al., 2020). The development of digital competences is necessary both for the academics to take advantage of the opportunities offered by technological advancement and to create strategies for their professional development. It is also necessary so that academics can help the improvement of the digital competence of the students themselves.

Many countries are currently in the process of developing or revising digital competence frameworks, self-assessment tools and training programs to guide teacher training and continuous professional development in this area. There are several initiatives, models and frameworks for assessing the digital competence of teachers, such as the ICT competences (Costa et. al., 2008), C2i2e Referential (Loisy et al, 2012) and the ICT skills and standards for the teaching profession (Hinostroza et al., 2009). In this study, we focus on the *DigCompEdu Framework*, widely used in the countries of the European Union and beyond.

The objective of DigCompEdu is to promote citizens’ learning of digital skills in member countries of the European Community and to enhance innovation in education (Redecker, 2017). The framework intends to capture and describe educators’ digital competences by means of 22 questions within a progression model organized into six different stages, allowing the educator to identify and gradually improve their competences. The competences are grouped into six areas that focus on different aspects of educators’ professional activities: (Area 1) Professional Engagement, (Area 2) Digital Resources, (Area 3) Teaching and Learning, (Area 4) Assessment, (Area 5) Empowering Learners, (Area 6) Facilitating Learners’ Digital Competence.

In this study we investigate the academics’ perceptions of their own digital competence in seven Iberoamerican countries. This is part of the activities of Metared, a collaborative network of higher education institutions which aims to study and disseminate the use of ICT in teaching and learning processes, as well as good practices in IT management and governance. Our results were obtained from universities in Argentina, Brazil, Colombia, Chile, Peru, Mexico and Portugal, based on the self-reflections of more than 30,000 academics.

On the subject of what the current literature says about digital competence development of academics in higher education, one can come across a prolific field of research. BasillotaGómez-Pablos et al.(2022) there has been a notable and exponential increase in the number of publications on TDC especially in recent years. The accounts found in the literature, although often based on relatively small localised samples, can together help drawing the landscape of the field.

Since we have collected data from a diverse population of academics in terms of gender, age, areas of knowledge and geographical location, we have decided to explore what our data revealed in relation to current research in the field, from the studies that have used the DigCompEdu framework or other instruments to analyse and discuss digital competences.

In terms of digital competence level, a study carried out by ), in which 300 academics from health sciences were surveyed in Andalucía, Spain, revealed that their level of digital competence was basic-intermediate, falling within levels A2-B1 as per the DigCompEdu framework. Another study carried out by Benali et al., (2018), with 160 academics from the field on English language teaching in Marruecos, showed that most academics fall in the intermediate level, B1-B2. Yet another study, carried out by Dias-Trindade et al., (2020) in a Portuguese university with 118 academics, revealed that their digital competence level was B1 (intermediate). We note that the levels of digital competence of these academics varied considerably, from A2-B2, and within this range there are many competence differences that are nuanced. We therefore proposed our first research question on the digital competence level of academics in our study, since we had such a large data set: Q1.*:* What is the digital competence of the academics in these Iberoamerican countries, as shown by the results of the self-reflections using the Check-In tool? Our larger sample size in relation to these studies fill in a gap identified by Cabero-Almenara et al. and Días Trindade et al., with authors agreeing on the need for future work based on larger sample sizes. ) also suggested that studies with an international perspective should be carried out, and our study also fills in that gap. In addition, we have proposed a question in relation to the process of going through a self-reflection, with the aim to see if the academic’s perceptions of digital competence have changed before and after answering the questions: Q.2 *What are these academics’ perceptions of their own digital competence before and after going through the self- reflection process using the Check-In tool?*

The higher education institutions also have a role to pay in relation to the use of digital tools by the academics. For example, the digital infrastructure of the institution and the level of support given to the academics are seen as variables related to a favourable perception for the integration of digital tools in the classroom (Fox et al., 2020; Tartavulea et al., 2020). From this we propose our third research question: Q.3 *How does the technological infrastructure offered by the participating Iberoamerican institutions affect academics’ digital competence?*

Furthermore, there seems to be controversy in the literature that certain factors tend to influence academics’ level of digital competence—such as their age and gender; their attitude towards the use of ICT; years of experience; level of training and seniority; and the quality of the infrastructure and other resources available at their educational institutions (Cabero-Almenara et al., 2020; Dias-Trindade et al., 2020; Guillén-Gámez et al., 2021).

According to Nwankwor (2021) and Dias-Trindade et al. (2020) the level of digital competence is not influenced by age. It is also said not to be influenced neither by gender (Basantes et al., 2020; Benali et al., 2018; Cabero-Almarana et al., 2021b; Guillén-Gámez et al, 2021; Nwankwor, 2021) nor by the length of experience of use of TICs (Nwankwor, 2021). However, other studies provide a different view. Guillén-Gámez and Mayorga-Fernández (2019) found differences in terms of age and years of experience and suggested that these variables worked against the development of digital competence. In this study, academics below 40 years of age and those below 10 years of experience presented a higher level of competence. In the same perspective, Benali et al., (2018) found that academics with more years of experience had better digital competence. On the other hand, Cabero-Almanara et al. (2021a) found that there are no significant differences in terms of gender, but the results suggested that there are differences between the level of digital competence and age, years of teaching experience and time of using TICS. By contrast, Basantes et al. (2020) have shown that the level of digital competence does not depend on gender, but on age: the higher the age, the lower the digital competence level. In addition, Hamalainen et al., (2021) suggest that there are two large groups of variables which seem to influence the integration and use of digital tools by academics: personal characteristics and contextual characteristics. From these current understandings we propose the research questions 4 and 5: *Q.4. Does the age of academics affect their level of digital competence ? And Q.5. Does the gender of academics affect their level of digital competence?*

Lastly, it seemed relevant to look at those competences that have the lowest and the highest scores, to elicit information that is helpful for the making of institutional policies on professional development of academics, and for the academics themselves to pursue their own routes for competence improvement. In this sense, Zhao et al., (2021) argue that there are many studies on digital competence focusing on undergraduate higher education students, but there is a lack of studies on digital competence focusing on teaching faculty—and our study contributes to filing that gap. We therefore propose the final three research questions: Q.6 What are the competence areas and topics where academics have the lowest levels of development? and Q7. What are the competence areas and topics where academics have the highest levels of development? Q. 8. Based on the global results of these self-reflections, what are the opportunities for institutional policy development by the higher-education institutions, aiming at enhancing the digital competence of the academics?

With this study our primary goal is to identify weaknesses and strengths in the digital competences of the surveyed academics, including the understanding of whether there was any influence of age, gender and- institutional infrastructure conditions. The results obtained for this sample of countries are also discussed in order to help the elaboration of guidelines or recommendations on how higher education institutions can develop institutional policies to improve the digital competences of the academics.

To address these goals, this paper is organized into five sections, beginning with this introduction. “Methodology” section presents the methodology and “Data analysis” section brings the data analysis. “Discussion” section contains a discussion of the outcomes, with a focus on aspects related to the development of institutional policies. ”Limitations of the study” section shows the limitations of the study and “Conclusions” section consists of conclusions and considerations.

## Methodology

In this section we present the research model and procedure, as well as the context and sample. Moreover, the DigCompEdu framework and the Check-In self-reflection tool are explained in more detail.

### Research model and procedure

The DigCompEdu framework report was produced in 2017 by the Joint Research Centre (JRC), the in-house research institute of the European Commission, in collaboration with the Directorate General for Education and Culture (DG EAC). It has been published in the format of a research report, aiming to inform policymaking in Europe. However, due to the immediate interest in the framework by many EU Member States (Caena & Redecker, 2019), it became evident that the framework would be most suited for immediate use if accompanied by an appropriate tool for its implementation. The so-called ‘Check-In’ tool was subsequently proposed by the JRC. The Check-In tool is based on the existing EU Survey platform of the European Commission. It is an online survey-management system which is free of charge to use and available in 23 languages. In essence, ‘Check-In’ is a nickname created by the JRC for the DigCompEdu framework applied to the platform in the format of a survey.

The transition from a theoretical framework to a self-reflection tool, in the form of questions available in the survey platform, was initially experimental. (Caena & Redecker, 2019, p. 364) argue that the development of the Check-In tool at the conceptual level was guided by three principles: (i) the need to condense and simplify the key ideas of the framework; (ii) the need to translate competence descriptors into practices that teachers could recognize, and (iii) the need to offer feedback to teachers in accordance with their individual levels of competence, with tips on how they could improve.

The self-reflection questions were developed in-house by the JRC, alongside external collaborators, by using the six areas of the framework as a basis for generating the questions. The need for a progression level was also evident, so the questions were assigned points per area, based on the levels A1–C2 as proposed in the framework. As a result, a master document was created, so that the questionnaire could be translated into different languages by interested parties, and its specific words changed on demand. This was to ensure the self-reflection was suitable for adaptation by users whose interests cover a range of education levels, including basic education, secondary education, higher education and adult education. Although created on an experimental basis, the Check-In tool became very popular, and is now used not only in Europe but also elsewhere. To date, it has had more than 100,000 users in education sectors across the globe.

### The proficiency model in the Check-In tool

The A1–C2 proficiency model offered by the tool is based on the Common European Framework of Reference for Languages (CEFR) and was developed to “help educators understand their personal strengths and weaknesses” (Redecker, 2017). The ‘A’ levels refer to the beginning of the journey by an educator, when they are starting to become digitally competent. A1 is the level of initial awareness, and A2 an exploratory level. B1 is the ‘integrator’ level, where users are expanding their practices, and B2 is the ‘expert’ level where users are already considered digitally competent and are enhancing their professional practice strategically. The ´C’ levels are higher levels of competence, with an academic at level C1 referred to as a ‘leader’, and at C2 as a ‘pioneer’. DigCompEdu presents specific skills and attitudes that users of the tool should be able to identify in their practice via the self-reflection, in each of the six areas of the framework: professional engagement, digital resources, teaching and learning, assessment, empowering learners, and facilitating learners’ digital competence.

By completing the self-reflection questions, the Check-In tool system provides tips on how to ‘level up’, so that an educator who is placed, for example, on level A2 in any given area of the framework should aim to move up to the next level, in this case B1. Tips on how to do so are offered automatically via a final report that covers each area of the framework and is issued to the user at the end of the survey.

The process of validating these proficiency levels took place throughout the development of the DigCompEdu framework, all the way to the experimental exercise of creating the Check-In tool as a self-assessment instrument. The development of the framework was first based on extensive desk research, where more than 50 instruments were identified as relevant and had their components analyzed (Caena & Redecker, 2019). Next, “32 of these were selected as basis for the generation of a first draft of the framework, which then were mapped to form competences and clustered to form competence areas”(Caena & Redecker, 2019, p. 362). Finally, the validation of the proficiency levels followed the desk research by the promotion of a series of stakeholder consultations, both face to face and online, which resulted in further versions of the framework. The stakeholders involved were teachers, researchers and policymakers. There have also been open online stakeholder consultations on subsequent drafts of the framework. Caena and Redecker (2019) argue that while these consultations were taking place, a consensus was reached by policymakers and other stakeholders on the need for the framework to include aspects of innovation underlying European education-policy initiatives, rather than being only a synthesis of other frameworks. It was therefore proposed that DigCompEdu should be not only a common reference for local European initiatives fostered by Member States but also a framework for policymaking with regard to educators’ digital competence across the European Union as a whole.

The Check-In self-assessment tool has also been produced via an iterative process of expert consultations, pre- piloting and items revisions (Benali et al., 2018; Caena & Redecker, 2019; Ghomi & Redecker, 2019). During these various processes the experts decided to map onto the framework the answer options for each of the suggested self-reflection questions. They also agreed to focus on what they considered the most basic competence within each item. In this sense the Check-In tool operates at the most basic competence level as decided by the experts, not encompassing all the elements discussed in each of the sub-items of the areas in the DigCompEdu framework.

The Check-In tool was designed to work on a points system, with the total possible number of points scored ranging from 0 to 88. Each question is multiple choice, with five answer to choose from. Depending on the answer selected, between zero and four points can be scored on each question. Here is the relationship between the number of points and the level of competence:

- Less than 20 points: Newcomer (A1)
- Between 20 and 33 points: Explorer (A2)
- Between 34 and 49 points: Integrator (B1)
- Between 50 and 65 points: Expert (B2)
- Between 66 and 80 points: Leader (C1)
- More than 80 points: Pioneer (C2)

Caena and Redecker (2019) argue that further modifications to the instrument will be made once data from different languages and sector versions of the tool are gathered and analyzed. This is because the Check-In tool has been built to work transversally in all education sectors by using different versions for each sector. Depending on the sector, changes at the semantic level have been proposed (the use of teachers for those employed in schools and academics or lecturers for those in higher education). The testing of the Check-In tool in schools took place first in Germany and has since been done by its own users in other EU Member States, as well as outside the EU. For example, the tool was tested in Portugal by Dias-Trindade et al., (2020) by analyzing the internal consistency of the items based on a calculation of the Cronbach alpha coefficient and an analysis of the construct validity via exploratory and confirmatory factorial analysis. The overall result indicated good validity, fidelity, and interpretable factorial structures. The tool has also been validated by Núñez-Canal et al., (2021), who have analysed 251 responses from a convenience sample of academics from universities in Madrid, in business administration; and by Cabero-Almenara et al., (2020), where the self-reflection was carried out with academics from Andalucía, Spain.

Nevertheless, the tool is in constant evolution, as foreseen by Caena and Redecker (2019), and a revised instrument has been launched in 2021 for the higher-education sector in the Check-In tool.

### Research context and sample

Metared is an initiative of the Universia Foundation, promoted by Santander bank as part of its programmes. It is a collaborative project, consisting of a network of networks, constituted by the Ibero-American Universities. Metared comprises universities in different countries and regions, namely Argentina, Brazil, Central America and the Caribbean, Chile, Colombia, Ecuador, Spain, Mexico, Peru and Portugal. Part of Metared’s mission is to promote national and international collaboration between universities in the field of information and communication technologies, and to support the digital transformation of universities.

The collaboration between the JRC and Metared, in order to introduce the Check-In self-reflection tool in universities in Latin American countries, dates back to October 2019. Its importance in terms of territorial scope and potential for informing policymaking in Latin America is inestimable. Metared invited the universities that are part of its network to carry out the study in their respective countries, with one university in each country responsible for local coordination of the study. A total of seven countries participated in this first round: Argentina, Brazil, Chile, Colombia, Mexico, Peru and Portugal. The time frame in which the research took place was from 1 October 2020 to 7 May 2021. The instrument used—that is, the self-reflection question^1^—had been translated into European Spanish by the Conference of Rectors of Spanish Universities (CRUE), but each of the participating countries in Latin America adapted the language and main concepts to their own needs, thus having a self-reflection instrument that would be appropriate to their contexts.

Within this timeframe of seven months, each country ran their self-reflection within a specific time range varying from four to six weeks. An important factor in this time frame is that the COVID-19 pandemic had been officially announced by the World Health Organization in March 2020. It should therefore be expected that the results of this study are different from what they would have been in more normal circumstances, with the pandemic having caused an unprecedented shift of teaching and learning practices towards the online mode. This study did not focus on measuring these changes, although it is likely they have been accelerated. On one hand, the relevance of this study is that it presents results that are contemporary regarding the digital competence of academics. On the other hand, the pandemic has shown that a study like this one is even more relevant and necessary than before.

### Instrument used and its validation

The research method used in this study was survey research. The survey method was enhanced by strong international collaboration, underpinned by Metared’s efforts towards the formation of universities’ networks, both country-based and cross-border. Starting from the university hosting the central coordination of the Metared network in each of the seven countries, open calls for participation were established, after which the data was collected by convenience sampling. In addition, all universities could participate, whether or not they were part of the Metared network. The data was collected using the European Commission’s Check-In tool and stored anonymously in the European Commission’s servers under the safeguard offered by the European General Data Protection Regulation (GDPR). The data was passed onto Metared’s coordination centre by the JRC at Metared’s request, and then distributed to the participating universities. Peru and Mexico had their data stored in the servers of Fundación Universia (Metared), also in accordance with the GDPR. This is because they were the last two countries to run the self-reflection survey, and by then a new and more powerful technological platform to run the survey had been developed by Fundación Universia, replacing the use of the Check-In tool with regard to this joint JRC–Metared initiative. This change of technological platform was in the back end and was not visible to users. However, the instrument used (the self-reflection questions) was the same as in the other countries, except for the addition of a module on ´open education’ based on the OpenEdu framework (Inamorato dos Santos et al., 2016). For the sake of this study the data of this open education module was not analysed. The data collected and stored in both servers was then integrated into a unique database, cleaned for ´test entries’ and made available to this research team for analysis. The database management system used to store the unified database was a SQL server 2019. The tool used to clean and transform the data into a homogenous form ready to be analysed was Visual Studio 2019—Integration Services. The tool used for the data analysis was PowerBI.

Once the data was analysed, the results obtained have been looked at against the results from other research studies presented in the introduction, where there was some controversy in terms of the influence of age, for example. From this engagement with previous research, and based on the need to understand how the data could help drive policies to improve the digital competence of academics, eight research questions have been formulated for this study, as presented in “Introduction” section.

The answers to these questions, according to the analysis of the data collected, will be presented in the next section.

## Data analysis

**Fig. 1:**
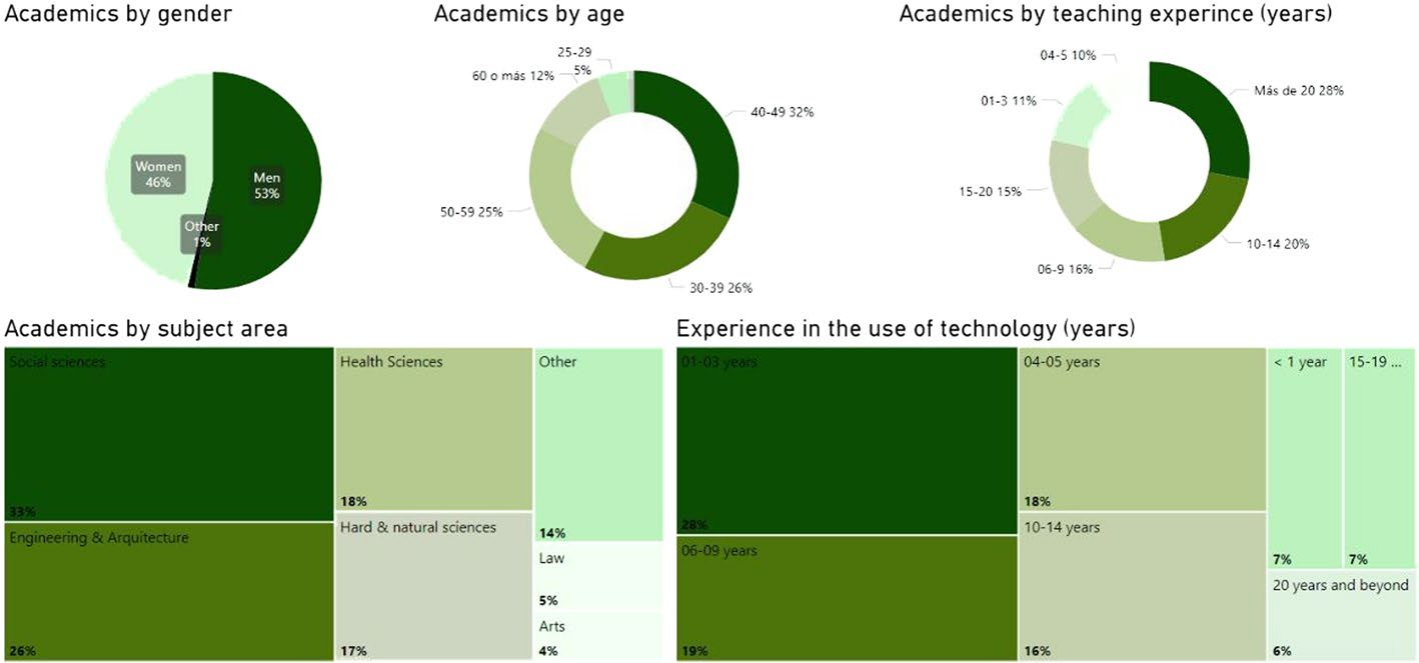
General dashboard—profile of survey participants

This section presents the results obtained, along with an initial data analysis. Since the data gathered in this study was not based on a representative design of the sample of the lecturers from these seven countries, but mostly out of their free will to participate in the self-assessment, it seemed important to verify whether the 30,407 lecturers who responded had any sort of bias in relation to age range, gender, years of experience, subjects taught, or length of experience in using technology. By analyzing these variables, no obvious bias has been detected. In fact, a wide variety of profiles have been identified, which makes it possible to attribute to the data a reasonable level of validity and reliability. From the graphic of the Fig. 1 it is possible to see the distribution of the lecturers in relation to these different variables.

Table 1 shows the number of participants per country and the average score obtained from the answer to the 22 questions of the DigCompEdu framework.

**Table 1:** Number of participants per country and their average score

Country	#Academics	Score
Argentina	3469	52.15
Brazil	2834	50.77
Chile	4084	53.32
Colombia	1396	55.67
Mexico	11,383	56.22
Peru	6540	58.83
Portugal	701	51.35

The 30,407 academics who participated in the survey belong to 403 different higher education institutions in seven countries. At the end of the self-reflection process the Check-In tool divided the academics into categories according to their perceptions of their level of digital competence, as shown in Table 2. For the purpose of a macroanalysis they were also grouped into three levels of performance: basic (fewer than 34 points), intermediate (between 34 and 65 points) and advanced (more than 65 points).

**Table 2:** Digital-competence level according to the results of the Check-In tool

Competence Levels	#Academics	%
A. Basic	1878	6.18
A1. Beginner	174	0.57
A2. Exploratory	1704	5.60
B. Intermediate	21,040	69.19
B1. Integrator	8665	28.50
B2. Expert	12,375	40.70
C. Advanced	7489	24.63
C1. Leader	6459	21.24
C2. Pioneer	1030	3.39

**Fig. 2:**
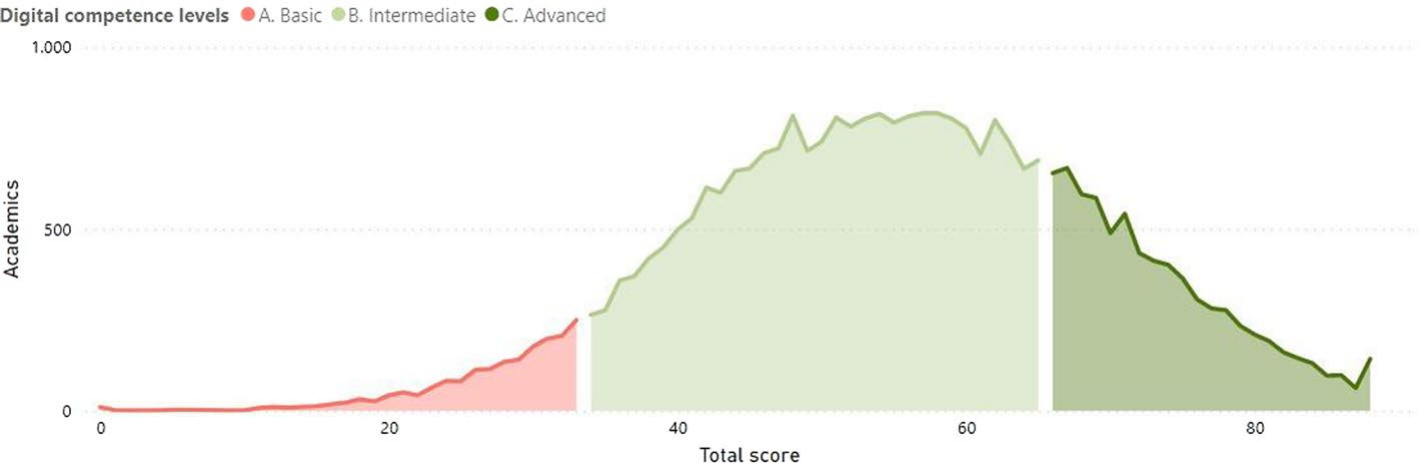
Digital-competence levels according to its score

Table 2 shows that 93.82% of the academics have either an intermediate or advanced level of competence, with 69.19% at intermediate level (B) and 24.63% at advanced level (C). Figure 2 illustrates the scores obtained by the academics.

The remainder of this section is devoted to other dimensions of analysis. “Level of digital competence according to initial and final perceptions” section examines academics’ levels of digital competence according to their initial and final perceptions (research question 2). “Relationship between work environment, age, gender and perceived level of digital competence" section looks the personal and institutional variables with the greatest influence on the results (research questions 3, 4 and 5). Finally, “Areas of digital competence with the highest and lowest levels of development” section presents the areas of digital competence where the academics perceive that they have the highest and lowest levels of development (research questions 6 and 7).

### Level of digital competence according to initial and final perceptions

This section presents how the academics are distributed according to their perceptions of their own digital competence. This self-reflection took place at two points in time: before the academics knew what the 22 questions in the Check-In tool were, and afterwards. Table 3 shows the results for both.

**Table 3:** Academics’ levels of digital competence, according to their initial and final perceptions

	Initial perception		Final perception	
Competence Levels	#Academics	%	#Academics	%
A. Basic	6168	20.28	5325	17.51
A1. Beginner	1333	4.38	905	2.98
A2. Exploratory	4835	15.9	4420	14.54
B. Intermediate	19,678	64.72	20,193	66.41
B1. Integrator	12,496	41.10	12,710	41.80
B2. Expert	7182	23.62	7483	24.61
C. Advanced	4561	15.00	4889	16.08
C1. Leader	3221	10.59	3726	12.25
C2. Pioneer	1340	4.41	1163	3.82

Firstly, it is important to highlight that the levels at which academics are classified by the instrument are the result a process of self-reflection. Initially this is based only on their own perceptions of their digital competences, and then later it is guided by the 22 questions that make up the DigCompEdu framework.

As can be seen in Table 3, there is a strong coherence in lecturers’ responses regarding their initial and final perceptions, either when evaluated by the six levels of the DigCompEdu progression model or when analyzed only three large groups: basic, intermediate and advanced. A clear majority—79.72% before using the 22 questions and 82.49% after—classify themselves at either intermediate or advanced level.

Another analysis that was carried out looks at the changes that occurred between the academics’ initial (rows) and final (columns) perceptions of their digital competences. This is illustrated in Table 4.

**Table 4:** Changes in digital competences according to the initial (rows) and final (columns) perception (all values are in %)

Final	A. Basic			B. Intermediate			C. Advanced		
Initial	A1. Begi	A2. Expl	Total	B1. Inte	B2. Expe	Total	C1. Lead	C2 Pion	Total
A. Basic	13.10	51.36	64.46	32.49	1.93	34.42	0.70	0.42	1.12
A1. Beginner	44.71	34.73	79.44	17.55	1.88	**19.43**	0.60	0.53	1.13
A2. Exploratory	4.38	55.95	60.33	36.61	1.94	**38.55**	0.72	0.39	1.12
B. Intermediate	0.40	6.12	6.51	53.17	33.68	86.85	6.01	0.63	6.64
B1. Integrator	0.54	8.79	9.33	72.76	15.02	87.78	2.47	0.42	2.89
B2. Expert	0.14	1.48	1.62	19.09	66.14	85.23	12.16	1.00	13.16
C. Advanced	0.42	1.05	1.47	5.33	16.16	21.49	54.83	22.21	77.04
C1. Leader	0.43	1.02	1.46	6.05	19.06	**25.12**	68.27	5.15	73.42
C2. Pioneer	0.37	1.12	1.49	3.58	9.18	**12.76**	22.54	63.21	85.75
TOTAL	2.98	14.54	17.51	41.80	24.61	66.41	12.25	3.82	16.08

Highlighted in bold are the largest displacements found in this study, occurring from Basic to Intermediate levels, besides the displacement from Advanced to Intermediate levels

Each line in Table 4 shows the movement of the academics who initially perceived themselves to be in one of the levels in the DigCompEdu progression and changed their perception after the self-reflection process, sometimes even putting themselves in a different category. Table 4 shows, for example, that 34.42% of those who classified themselves at basic level and 21.49% of those who classified themselves at advanced level changed their perceptions and re-evaluated themselves at intermediate level. Note that only 1.12% of the academics who evaluated themselves as basic level at the beginning of the evaluation process changed their perception and believe they are at the advanced level after completing the questionnaire. In general, the direction of these changes in perception was towards the intermediate level, given that most lectures are classified at the integrator and expert levels.

### Relationship between work environment, age, gender and perceived level of digital competence

Academics were asked the following questions related to the work environment offered by the institutions:

- 1. Does the university promote the integration of digital technologies in teaching?
- 2. Does the university invest in updating and improving technical infrastructure?
- 3. Does the university provide necessary technical support?
- 4. Do students have access to digital devices?
- 5. Is the university’s Internet connection reliable and fast?
- 6. Are interactive whiteboards, projectors or similar presentation media available in the classrooms you teach in?

The answers were given on a Likert scale, in which the participant should select one of the five options that best expresses their opinion in response to the question. The options are ‘strongly disagree’, ‘disagree’, ‘neither agree nor disagree’, ‘agree’ and ‘strongly agree’.

In order to understand whether the decisions taken by the institutions positively influence the competences of the academics, four of the previous six questions are analyzed and grouped into two questions, as follows:

- The institution invests in ICT infrastructure, such as:
- Is the Internet connection reliable and fast?
- Are interactive whiteboards, projectors or similar presentation media available in the classrooms you teach in?
- If the institution offers technical support:
- Does the university promote the integration of digital technologies in teaching?
- Does the university provide the necessary technical support?

By crossing the results of each question, it was found that these items have contributed to raising the levels of competence of the academics. With regard to the academics’ perception of the ICT infrastructure, shown in Table 5, it is noted that the ones who answered ‘strongly agree’ in both variables attained the highest averages (61.90, corresponding to 5,873 academics).

**Table 5:** Relationship between technology investment criteria and average score

Fast and reliable internet connection	1. Str. Agree		2. Agree		3. Neutral		4. Disagree		5. Str. Disagree	
Use of interactive whiteboards and projectors	Score	#acad	Score	#acad	Score	#acad	Score	#acad	Score	#acad
1. Strongly agree	61.90	4873	58.11	2949	56.98	1219	57.30	396	56.31	185
2. Agree	56.53	2062	54.08	5211	52.93	2546	53.05	1166	55.04	451
3. Neutral	54.19	517	52.75	1386	50.86	1856	52.07	721	52.19	380
4. Disagree	52.73	171	50.59	594	50.18	673	52.20	800	52.83	420
5. Strongly disagree	52.12	91	51.63	241	51.96	336	52.63	452	52.61	710

#acad means number of academics; Str. means "Strongly"

In relation to perceptions of the support provided by the institution (Table 6), the 10,000 academics who indicated that they ‘agree with the support received and the promotion of ICT integration in my institution’ reached the highest averages (59.73).

**Table 6:** Relationship between technical-support criteria and average score (#acad means number of academics; Str. means "Strongly")

Provides technical support	1. Str. agree		2. Agree		3. Neutral		4. Disagree		5. Str. Disagree	
Promotes technology integration	Score	#acad	Score	#acad	Score	#acad	Score	#acad	Score	#acad
1. Strongly agree	59.73	10,657	56.60	4053	57.25	988	57.66	276	61.08	77
2. Agree	52.66	1096	51.93	5972	52.54	2419	51.90	860	50.21	200
3. Neutral	50.16	93	47.41	578	48.53	1125	49.26	543	51.99	186
4. Disagree	51.92	12	44.67	89	45.75	123	49.61	348	50.20	178
5. Strongly disagree	58.25	8	55.78	23	52.73	40	53.48	80	53.53	382

#acad means number of academics; Str. means "Strongly"

When the average result is evaluated against the four variables as ‘highly agree’, the points obtained are the highest ones (62.87). The age and gender variables were also tested and validated to provide further evidence to the age controversy identified in the literature and presented in “Introduction” section. The results can be seen in Table 7.

**Table 7:** Relationship between age, gender and average score

Gender age	Male %acadScore		Female %acadScore		No answer & others %acadScore		Total %acadScore	
Under 24	0.25	51.00	0.58	56.84	0.01	33.33	0.84	54.80
25–29	2.12	56.91	2.67	56.35	0.03	36.44	4.82	56.47
30–39	12.56	56.70	12.99	55.85	0.17	54.33	25.72	56.25
40–49	16.17	55.99	15.17	55.77	0.19	53.17	31.53	55.87
50–59	13.44	54.48	10.90	55.36	0.13	52.00	24.46	54.85
Over 60	7.91	51.55	3.79	53.43	0.04	51.85	11.74	52.16
Rather not answer	0.22	51.29	0.22	55.55	0.43	53.05	0.87	53.25
Total	52.67	55.10	46.32	55.55	1.01	52.42	100.00	55.28

%acad means percentage of academics

The average score ranged from 52.16 points (academics aged over 60) to 56.47 points (academics aged 25–29). This variation is too small to have a significant influence. As for the gender factor, women attained an average score of 55.55 and men 55.10, in which case there is no influence found.

### Areas of digital competence with the highest and lowest levels of development

In this last category of results, it is shown how the academics are distributed according to the six areas of competence of the DigCompEdu framework. This is shown in Table 8 and represents the results obtained by the Check-In tool.

**Table 8:** Percentage of academics by area according to the DigCompEdu proficiency model

	Area 1	Area 2	Area 3	Area 4	Area 5	Area 6
Competence levels	Professional engagement (%)	Digital resources (%)	Teaching and learning (%)	Assessment (%)	Empowering learners (%)	Facilitating learners’ digital competences (%)
A1. Beginner	2.18	3.59	3.13	3.77	5.53	8.56
A2. Exploratory	14.83	14.13	11.20	16.26	16.44	10.89
B1. Integrator	37.17	31.68	28.37	29.87	24.39	36.99
B2. Expert	34.23	33.23	36.92	30.48	25.02	31.03
C1. Leader	9.86	14.78	15.11	14.62	20.08	9.95
C2. Pioneer	1.72	2.59	5.28	5.01	8.58	2.59

The general scenario presented in Table 8 shows that more than 70% of the academics are at the intermediate or advanced levels of the proficiency model, regardless of the area of digital competence. In competence areas 1, 2 and 3, for example, the percentage is higher than 80%. Areas 3, 4 and 5—Teaching and Learning, Assessment, and Empowering Learners—are those with the highest percentages of academics at advanced levels, with 20.39%, 19.63% and 28.66%, respectively. These areas directly concern the didactic and pedagogic competences of the academic, one of the dimensions of the DigCompEdu framework.

On the other hand, as the data is presented in the form of average results, it is important to analyze each one of the digital competences individually, observing and discussing their particularities. Therefore, the ten digital competences with the worst and best results in terms of score will follow.

Table 9 shows the ten worst digital competences and their areas in terms of score. Specifically, Table 9 shows the questions with the highest number of responses with a score of 0. A list of all DigCompEdu competences is presented in Appendix 1.

**Table 9:** The ten digital competencies with the highest number of academics who scored 0

Competence	Area	#Academics	%
5.2 Differentiation & Personalisation	Area 5	2915	9.59
2.3 Managing, protecting and sharing	Area 2	2890	9.50
6.4 Responsible use	Area 6	1889	6.21
1.2 Professional collaboration	Area 1	1403	4.61
6.3 Content creation	Area 6	1324	4.35
3.3 Collaborative learning	Area 3	1228	4.04
6.1 Information &media literacy	Area 6	941	3.09
3.4 Self-regulated learning	Area 3	890	2.93
6.2 Communication	Area 6	777	2.56
6.5 Problem solving	Area 6	739	2.43

The competence Differentiation & Personalisation—belonging to Area 5, Empowering Learners—presents the lowest score in the entire study, with 9.59%. If analyzed from a scoring perspective (0 and 1), more than 35% of academics have difficulties in the development of this competence.

Table 9 also reveals an interesting aspect of Area 6, Facilitating Learners’ Digital Competence: all five digital competencies assessed in this area are present among the ten with the lowest scores. Again, expanding the analysis to scores 0 and 1, Area 6 has three digital competences among the ten with the lowest scores: Responsible Use, Information and Media Literacy, and Content Creation.

Almost one-third of all academics surveyed (30.55%) scored zero for at least one of the 22 digital competences analyzed by DigCompEdu. This percentage increases to 70.51% when we consider scores 0 and 1, corresponding to more than 21,000 academics.

The best scores obtained by the academics and their corresponding digital competences were also observed in the DigCompEdu Framework. This is shown in Table 10, with the number and percentage of academics who achieved the highest score associated with the highest level in the progression model.

**Table 10:** The ten digital competencies with the highest number of lecturers who scored 4

Competence Levels	Area	#Academics	%
5.1 Accessibility & Inclusion	Area 5	11,194	36.81
1.4 Digital CPD	Area 1	10,394	34.18
3.2 Guidance	Area 3	9250	30.42
3.3 Collaborative learning	Area 3	8235	27.08
5.3 Actively engaging learning	Area 5	6063	19.94
3.1 Teaching	Area 3	5645	18.56
5.2 Differentiation & Personalisation	Area 5	5552	18.26
4.1 Assessment strategies	Area 4	5390	17.73
6.3 Content creation	Area 6	4862	15.99
2.3 Managing, protecting and sharing	Area 2	4634	15.24

When analysing the first two competences in Table 10, obtained by about 21,500 academics, it can be observed that they are concerned with their own development, through their participation in online training, as well as with students’ practical and technical difficulties. The latter may be associated with the fact that the research was carried out in 2020, after the beginning of the COVID-19 pandemic.

Finally, 22,553 academics have at least one digital competence with a score of 4, even if it is not among the ten shown in Table 10. This represents a significant proportion of the surveyed academics: almost 75%.

## Discussion

Eight research questions were proposed in “Introduction” section and were the focus of the data analysis. The overall goal as to understand the academics’ digital competence level, using the DigCompEdu framework, and within its 22 competences in 6 competence areas. Based on the study results, most of the academics (69.19%) perceive themselves to be at an intermediate level (B1–B2).

It is very likely that the inevitable experimentation with digital technologies for facilitating teaching and learning during the COVID-19 pandemic, due to the cancellation of face-to-face classes, was an important factor in these results, although there are no elements in this study that would aid a more detailed understanding. Apart from that, it is possible to suggest that we find ourselves at a moment of empowerment for educators. It is a particularly appropriate time to make changes and embrace the use of educational technologies in teaching and learning. It is also important to pay special attention to the use of pedagogical approaches that are adequate to the digital era. This is therefore a moment of change and advancement towards innovative strategies that include digital technologies in teaching, both in the face-to-face and the online mode. Likewise, for the 6% of academics at the A1 and A2 levels, it would be important to think of professional-development strategies to help them build their competences at a faster pace so that they can be included in the institutional strategies meant to advance the digital competence of most of the academics.

Regarding question 2, looking back at Table 4, it becomes evident that the perceptions of a significant percentage of academics (34%) changed after the self-reflection process in the Check-In tool. On the other hand, it is observed that the ones at the lower levels (A1–B1) tend to underestimate their digital competence while the more advanced (B2–C2) tend to overestimate theirs. However, it is noticeable that at the lower levels (A1–A2) this percentage is much higher, at around 50%, and corresponds to an underestimation of their digital competence. This is an interesting fact, since going through the self-reflection process seems to support the transformation of teaching practices in the sense that academics become more confident in and aware of their digital competence. In addition, in terms of the academics who do strengthen their self-confidence, the self-reflection process creates pathways for them to make an action plan and to advance their digital competence building.

For the reasons above it seems very important to strengthen the confidence of academics in their digital competences, at the same time as helping them improve. This could be done as part of institutional strategies, by promoting policies of digital transformation.

In “Relationship between work environment, age, gender and perceived level of digital competence” section of the results and in relation to the fourth and fifth research questions, which are about the influence of gender and age on academics’ digital competence, it was possible to revisit the level of influence of certain personal and institutional aspects on their digital competence based on the final points obtained. With regard to the personal aspects, the data in Table 7 shows how gender does not seem to have any incidence, since the results for men and women are nearly identical. In terms of age, it was expected that the younger academics would have a higher level of digital competence than their older peers. In this sense a correlation was indeed observed, with scores decreasing as age increases.

However, the biggest difference is that of 4.31 points between the average scores of academics aged over 60 and those aged 25–29. This does not seem to be particularly significant, however, as the only ones who do not follow the tendency are the ones who are aged 24 or below, since they have a lower average score than those aged 25–29. It is possible that the main factor contributing to their low scores is their relative lack teaching experience. This is an interesting finding, since it suggests that the digital competence of academics is not defined by a generation gap but more by a change related to the strategic use of technologies in the teaching process, which should be accompanied by the right choice of pedagogical practices.

In Tables 5 and 6 of “Relationship between work environment, age, gender and perceived level of digital competence” section—regarding the third research question, about the influence of the institutional context—it is shown that among the factors with a positive influence on academics’ competences is the IT support offered by their universities. Most academics believe that their institutions provide the necessary IT infrastructure and support them in the use of digital technologies. The results above can be used to inform the implementation of institutional and governmental policies that support investment in technological infrastructure and give adequate support to academics in terms of technology use.

With regard to the research questions 6 and 7, on the areas in which the academics perceive that they have the highest and lowest levels of digital competence, Tables 9 and 10 draw our attention to how the development of students’ digital competences has been promoted.

The analysis of the Table 9 makes clear that there are certain competences that most academics should further develop. Although 94% of the academics surveyed have intermediate or advanced digital competence, 70% of them nevertheless scored between 0 and 1 in at least one of the competences. This is particularly the case with the competence ‘Differentiation & Personalisation’. This reveals the challenge of providing different and additional tasks for students according to their learning profile. In addition, the Differentiation & Personalisation competence offers the opportunity for intensive use of digital techniques and tools in online and hybrid learning, such as those provided by artificial intelligence, pattern recognition and recommendation systems.

In addition, all competences in area 6, ‘Facilitating the Digital Competence of Learners´, could be further developed, since the data shows that its five competences are among the ten in which the academics scored lowest. For example, the competences ‘Responsible use’ and ‘Digital Content Creation’ are among the five with the highest number of academics who scored 0. This means there do not seem to be enough teaching activities that encourage students to express themselves digitally, and little attempt to empower them to identify and manage the risks of technology use, whether in terms of cyber security or physical and mental health. This is an important finding from this study. It suggests that if current students represent the technology-driven ‘digital natives’ of ‘Generation Z’ (aged between 18 and 23), the current generation of academics do not seem equipped to exploit those students’ potential.

Table 10 shows the ten digital competences in which academics considered that they were strongest, assigning themselves 4 points. In the first four competences, at least one-quarter of the academics (from 27.08 to 36.81%) gave themselves the top mark of 4 points. Those competences are 5.1, Accessibility and Inclusion; 1.4, Digital CPD (continuous professional development); 3.2, Guidance; and 3.3, Collaborative Learning. These competencies could serve as examples and targets in terms of making sure that most academics achieve similar competence levels, thus achieving a levelling-up process. At the same time, however, the results make clear that most academics do not possess such competences and would need to work towards acquiring them.

Finally, with regard to research question number 8, concerning opportunities for new institutional policies on the part of higher-education institutions, the results shed some light on where the focus should be when it comes to developing the digital competence of academics. Both the competences with the highest scores and those with the lowest scores help us understand where the digital gaps in higher education seem to be, both in terms of teaching and learning. In deciding which competences to develop further, institutions should also consider their pedagogical model.

Some of the competences with the lowest scores—for example 3.4, Self-Regulated Learning, and 5.2, Differentiation and Personalisation—could benefit from institutional strategies to empower learners towards autonomous learning, and the use of learning analytics and artificial intelligence to foster personalisation and differentiation in the learning process.

The competences with the highest scores—such as 5.1, Accessibility and Inclusion; 1.4, Digital CPD; and 3.2, Guidance—could reflect the efforts made during the COVID-19 pandemic to reach out to all students via online learning, and the efforts many countries and institutions had already been making in order to foster the modernisation of higher education via the use of digital technologies in teaching and learning processes. These efforts should continue, so that all academics receive the benefits.

Overall, the results show that digital competence means more than merely being able to operate certain technologies: it involves being able to use them in a meaningful way, underpinned by specific pedagogies, so as to achieve specific goals in addition to the teaching of the core subject. One example is helping learners to be more responsible for their learning processes, while at the same time empowering them to make informed decisions and therefore better choices in life. Digital competence for educators is above all related to how to teach, tapping into appropriate pedagogies while at the same time using technology to enhance the processes of teaching and learning.

## Limitations of the study

It is essential to interpret the results bearing in mind that the self-reflection process is based on subjective perceptions, not on an objective measurement of knowledge and skills. When we analysed and compared our results to other studies, we took into consideration only publications in English. It would also be advisable to discuss the results of further studies with policy makers via focus groups, for example, to identify the possible routes to take from the results obtained.

## Conclusions

This self-reflection process, carried out in seven Iberoamerican countries and involving more than 30,000 academics, was based on collaborative effort and teamwork. For the institutions that took part, its success depended on local efforts to mobilise the academics to respond to the self-reflection, making them aware of the need to better understand their level of digital competence. The knowledge gained as a result would appear to have been helpful both for the academics as individuals, via the feedback they have received, and for the institutions themselves, via the aggregated institutional data they received.

Although all the academics were free to choose whether to go through the self-reflection, the results are still very significant because they show the engagement of all collaborators in the seven countries in obtaining data that would enable the opening of areas of opportunity for the further development of higher- education policies in Iberoamerica. This exercise was carried out during the global COVID-9 pandemic, a fact that should be taken into consideration when looking at the data. It was a period when most activities in higher education had to be carried out online. It was therefore probably the case that almost all the participating academics were already having to use digital technologies in their teaching. What we do not know is whether the pedagogical approaches they were using for teaching online were the most appropriate for engaging with learners. The results suggest, however, that there is room for improvement.

Based on the results of more than 30,000 academics surveyed, the biggest sample carried out to date on the DigCompEdu-Check-In tool,, the answer to question 1 on the digital competence level of academics is that the academics’ average level of digital competence, according to their own perceptions, was ‘B’ or Intermediate—a range within which, according to the DigCompEdu framework, B1 is ‘Integrator’ level and B2 ‘Expert’. At B2 level an academic can experiment with digital technologies in a variety of ways and for a range of purposes, being able to use them confidently and creatively. However, B-level individuals are still working on understanding which tools work best, with many options not yet tried. These individuals are therefore in need of further professional development in digital competences, since these profiles, as per the DigCompEdu framework definition, are curious professionals, open to new ideas and to experimentation. In addition, it is important to consider that the results are not even for all areas of the framework, meaning that while B is the average level of competence of all academics, there are several competence areas which, if looked at separately, showed a very low score in the academics’ self-perception. This is the reason why, depending on the perspective taken, one might look at these B- level (intermediate) results under the perspective of ‘a glass half empty or a glass half full’. Previous studies have been similar in positioning academics in the B level. ) surveyed 300 health sciences academics from 9 universities in Spain and the result was basic intermediate. Días-Trindade et al. (2020) surveyed 118 academics from one Portuguese university, and the average result was B1 (Integrator). We went a step further in our data analysis to identify the ten digital competencies with the highest number of academics who scored 0, and the ten digital competencies with the highest number of academics who scored 4 (the maximum points). These are presented on Tables 9 and 10. Almost 1/3 of all academics surveyed scored 0 for at least one of the 22 digital competences analysed in the DigCompEdu. This is so that it becomes evident that the average score of the DigCompEdu—Check-In self-reflection should always be looked at in relation to the areas’ score, because otherwise it could be misleading and prevent appropriate actions towards professional development to take place.

Regarding our question 2, whether there was a change in the self-perception of academics about their digital competence before and after going through the self-reflection process, we have seen that the perceptions of a significant number of academics have changed (34%). This means that going through a self-reflection on digital competence is, per se, a way of learning about one own skills, therefore can be considered a valid process, despite of not being a full evaluation of competences. This result validates not only the framework, but also the self-reflection process as a useful tool for competence analysis.

Question 3, on how the technological infrastructure offered by the institution affect the perception of digit and Petre al competence, our results are aligned with that of Fox et al., (2020), and of Tartavulea et al. (2020), arguing that it affects favourably. The 4th research question, on whether the age of academics affect their digital competence, was meant to add further evidence to a current controversy in the literature where some studies argue that it has an effect whilst others do not think this is the case. Our study with a large sample of academics was able to identify some nuances to this matter. The assumption that younger academics had a better digital competence than their older peers seem to have been validated in our research, since we observed a correlation, and the scores decreased as the age increased. However, the biggest differences were observed with academics aged 60 + and academics between 25–29 years old. Even more important was the difference of the young academics aged below 24 years-old, because they have scored lower than the ones between 25–29 years old. In this sense, our research validates that age can have an impact on the digital competence of academics. However, we observed that age is not the only factor to be considered, but also experience. The academics aged below 24 years old scored lower due to having less pedagogical experience. A similar conclusion has been reached by ). In terms of research question 5, on gender, our results show that it does not have any influence on the digital competence of academics, thus hoping to contribute to livening up the controversies in the literature in this matter, as discussed in the introduction.

Our final three research questions are about the areas in which academics perceive they have the lowest level of digital competence, (Q6), and the highest (Q7). Finally, question 8 is about the identification of institutional policy development by higher education institutions, based on these results. For question 6 we have shown on Table 9 that the 10 topics with lowest scores. The competence Differentiation & Personalisation is the one where most academics scored 0, followed by Managing, protecting and sharing digital resources. For question 7, the areas with the highest score are Accessibility and Inclusion followed by Digital CPD. The full results are presented on Table 10.

Finally, for question 8, we enquired about the global results of these study and the opportunities for institutional policy development, we have reached several conclusions. The study showed how institutions can work collaboratively towards a shared goal through the efforts of the individuals they employ, taking advantage of the opportunity to promote self-awareness and institutional awareness and, above all, to provide policy support in the countries involved. Enabling staff to engage with a self-reflection process on their digital competence can help the institution identify the areas where professional development activities should focus on, and whether their infrastructure investments are paying off. All research questions of this study contribute to answering question 8. On one hand, the methodology of this study allowed universities to zoom into the institutional results, which were given to each institution anonymised and in an aggregated way. On the other hand, the global results, showing the scoring of all the institutions allow for the individual institutions to benchmark themselves. The large sample size we had (30.000 + academics) and its diversity in terms of the academics’ area of knowledge, age, gender and country of origin are unique to date, and the main strength of this study. A large sample size is more representative of the population, forming a better picture for analysis.

It is important to understand how these results could best support professional- development initiatives and assist higher-education institutions and governments in implementing policies conducive to improving digital competence. The results indicate that further support for academics is necessary and would therefore be welcome, both with regard to their own practices and how they could be most effective in improving the digital competence of their students. Enhanced digital competence would enable students to act in society as empowered citizens in respect to responsible technology use. A possible direction for future research could be to measure academics’ self-perceptions against their actual practices. For this to happen, a different frame of analysis would be needed, with a more objective means of evaluation. Another challenge deserving of further research is that of finding the most effective ways to translate the results into policies. Since training programmes are likely to be designed around the framework topics, there seems to be room for collaboration between institutions, regions and countries.

## Data Availability

The anonymized data set is available at https://www.metared.org/global/datosabiertos. For any queries on the data, please contact: comunicacion@metared.org.

## Appendix 1

### European framework for the digital competence of educators (DigCompEdu)

List of 22 digital competences of educators, organized in 5 (five) areas, considered relevant to innovate in education (Redecker, 2017).

Area 1—Professional Engagement

- 1.1 Organisational Communication
- 1.2 Professional Collaboration
- 1.3 Reflective Practice

- 1.4 Digital CPD

Area 2—Digital Resources

- 2.1 Selecting
- 2.2 Creating & Modifying
- 2.3 Managing, Protecting, Sharing

Area 3—Teaching and Learning

- 3.1 Teaching
- 3.2 Guidance
- 3.3 Collaborative Learning
- 3.4 Self-regulated Learning

Area 4—Assessment

- 4.1 Assessment Strategies
- 4.2 Analysing evidence
- 4.3 Feedback & Planning

Area 5—Empowering Learners

- 5.1 Accessibility & Inclusion
- 5.2 Differentiation & Personalisation
- 5.3 Actively Engaging Learners

Area 6—Facilitating Leaners’ Digital Competence

- 6.2 Communication
- 6.3 Content Creation
- 6.4 Responsible Use
- 6.5 Problem Solving

## Footnotes

1 Available at https://www.metared.org/content/dam/metared/pdf/Check_In_DigCompEdu_Self_Reflection_Tool.pdf
